# Solvent-Free and Scalable Procedure to Prepare PYR13TFSI/LiTFSI/PVDF–HFP Thermoplastic Electrolytes with Controlled Phase Separation and Enhanced Li Ion Diffusion

**DOI:** 10.3390/membranes9040050

**Published:** 2019-04-10

**Authors:** Víctor Gregorio, Nuria García, Pilar Tiemblo

**Affiliations:** Instituto de Ciencia y Tecnología de Polímeros, ICTP-CSIC, Juan de la Cierva 3, 28006 Madrid, Spain; v.gregorio@ictp.csic.es (V.G.); ngarcia@ictp.csic.es (N.G.)

**Keywords:** PVDF–HFP, solid electrolytes, thermoplastic, Li diffusion PVDF–HFP, solid electrolytes, thermoplastic, Li diffusion

## Abstract

Solid electrolytes for Li transport have been prepared by melt-compounding in one single step. Electrolytes are composed of polyvinylidene fluoride–hexafluoropropylene (PVDF–HFP) with PYR13TFSI on its own or with varying concentration of LiTFSI. While the extrusion of PVDF–HFP with PYR13TFSI is possible up to relatively high liquid fractions, the compatibility of PVDF–HFP with LiTFSI/PYR13TFSI solutions is much lower. An organo-modified sepiolite with D-α-tocopherol polyethylene glycol 1000 succinate (TPGS-S) can be used to enhance the compatibility of these blends and allows to prepare homogeneous PYR13TFSI/LiTFSI/PVDF–HFP electrolytes with controlled microphase separations by melt-compounding. The structure and morphology of the electrolytes has been studied by FTIR, differential scanning calorimetry (DSC), SEM, and AFM. Their mechanical properties have been studied by classical strain–stress experiments. Finally, ionic conductivity has been studied in the −50 to 90 °C temperature range and in diffusivity at 25 °C by PFG-NMR. These electrolytes prove to have a microphase-separated morphology and ionic conductivity which depends mainly on their composition, and a mechanical behavior typical of common thermoplastic polymers, which makes them very easy to handle. Then, in this solvent-free and scalable fashion, it is possible to prepare electrolytes like those prepared by solvent casting, but in few minutes instead of several hours or days, without solvent evaporation steps, and with ionic conductivities, which are very similar for the same compositions, above 0.1 mS·cm^−1^ at 25 °C. In addition, some of the electrolytes have been prepared with high concentration of Li ion, what has allowed the anion exchange Li transport mechanism to contribute significantly to the overall Li diffusivity, making *D_Li_* become similar and even clearly greater than *D_TFSI_*.

## 1. Introduction

All-solid state is a must in the future battery technologies mainly (but not only) because of safety reasons. Leaks and dendrite growth appear as major issues which have to be overcome, and it does not seem possible that liquid electrolyte batteries can completely eliminate both these flaws. On their turn, low conductivity and electrochemical stability, together with poor solid–solid interfaces, are the main drawbacks of all-solid-state batteries, and again there are no straightforward solutions for them.

An optimum electrolyte should completely avoid leaks and dendrite growth, withstand high voltages, allow rapid ion diffusion, provide excellent interfacial contact, and produce a performing solid electrolyte interphase (SEI); in other words, an optimum electrolyte should behave at the same time as liquid and as solid. This is a very challenging defy for material scientists, which can be successfully solved if the length scales at which the material has to behave as a liquid and as a solid are different. Inspiration can be found in the field of mass transport (e.g., gas or liquid separation with polymer membranes), which is a mature technology where similar challenges as in ion transport had to be undertaken decades ago. For instance, electrolytes composed of a polymer separator or a porous membrane soaked in a liquid electrolyte are analogous to porous membranes for mass transport. They will be solid at a macroscopic scale, and hence self-standing, flexible, light, tough, and as ion conduction will occur through the liquids in the pores, ion conductivity will be high and electrode wetting will be high. However, some of the requirements may not be fulfilled by this strategy, because these materials will be liquid at the length scale at which dendrites are produced [1,2], and they will also allow for quick reaction rates, chain reactions, and flammability. This means that separators soaked in a liquid electrolyte will not be safe enough for numerous applications.

For the sake of safety, some authors believe that the final goal would be an all-polymeric electrolyte without a liquid phase. It may be unnecessary to completely avoid the mixing of the polymer with an electroactive liquid phase if the final electrolyte blend displays specific mechanical requirements, which are still not completely clear, but which involve large enough shear modulus (as compared to that of the metal electrode) or plastic deformation [3,4]. Gel electrolytes composed of very large fractions of liquid phase, though not porous, are at the microscopic scale very close to being too similar to a liquid, and their elastic moduli are low, and hence dendrite growth will also occur. A dense (as opposed to porous) polymer membrane, composed of a polymer blended with a fraction of non-flammable safe liquid providing a composite material with a reasonable shear modulus seems to be a very worthy target not only for Li batteries but as a general approach, as dendrites are a safety issue in other metal cation batteries as well [5,6,7].

In the past, we have proposed thermoplastic electrolytes consisting of dense membranes composed of poly(ethylene oxide) (PEO) and electroactive phases, either the classical carbonates [8,9], or RTILs [10,11], which have the particularity of being prepared in the absence of solvents by melt-compounding. This is a very interesting preparation procedure, because solvent cast electrolyte preparation is extremely time-consuming and unsustainable. The concept has been developed for Li electrolytes but is also valid for Na or other cations. The mechanical properties of these melt-compounded thermoplastic electrolytes are excellent and their electrochemistry very reasonable, as they eliminate dendrites [12] and show promising cyclability [13].

Though for voltages up to ≈4 V [14] electrolytes prepared with PEO perform well, the high energy density Li metal batteries require electrochemical stabilities better than those that can be offered by PEO. Other polymer candidates are polymethylmethacrylate (PMMA) and the fluorinated copolymer polyvinylidene fluoride–hexafluoropropylene (PVDF–HFP). PVDF–HFP has for long been studied [15] because of its high electrochemical stability, excellent mechanical properties, and its high compatibility (even miscibility) with other compounds of interest in the design of electrolytes [16], such as PMMA or room-temperature ionic liquids [17].

PVDF–HFP based electrolytes are either solvent casted with electroactive liquid phases [18,19,20], or prepared in porous morphologies and soaked with the electroactive liquid phases [21,22]. The review of the literature [18,21,23,24] shows that common formulations of PVDF–HFP based electrolytes include high fractions of pyrrolidinium LiTFSI ionic liquids in which LiTFSI is dissolved, and as PVDF–HFP is able to incorporate large fractions of liquid phase, their conductivity is consequently high. It has to be taken into account that PVDF–HFP prepared by solvent casting in the presence of an ionic liquid phase produces dense or porous structure [22] depending on the preparation conditions. It is important to bear in mind that porous and dense membranes are in fact two very different types of materials. Even though their macroscopic appearance may be similar, their morphology and microstructure will be very different, and so properties that depend on them, for example, ion diffusion, which in turn governs not only ionic conductivity but also metal dendrite growth.

In this work, we propose an alternative to porous membranes soaked in electroactive phases:—dense membranes with phase segregated morphology, sharing advantages with porous soaked membranes and presenting lesser drawbacks. The electrolytes consist of blends of a pyrrolidinium ionic liquid, LiTFSI, and PVDF–HFP, which can be prepared by a solvent-free melt-compounding procedure, in only a few minutes, and which present ionic conductivity that can compete with similar electrolytes prepared by solvent casting or soaked PVDF–HFP porous scaffolds. Two strategies are employed to increase Li conductivity in these electrolytes:—controlled phase separation and promotion of Li anion exchange diffusion in addition to Stokes transport.

## 2. Materials and Methods

### 2.1. Materials

**Scheme 1:**
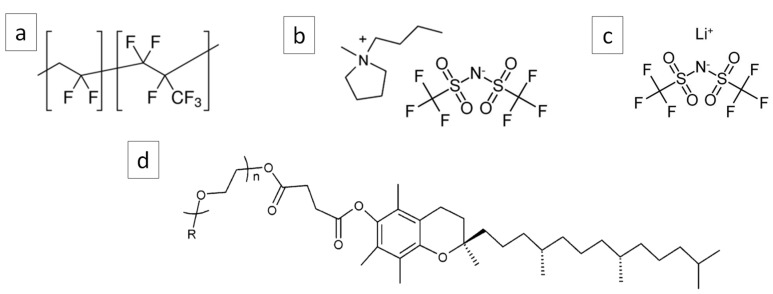
Chemical structures of the materials employed: (**a**) polyvinylidene fluoride–hexafluoropropylene (PVDF–HFP), (**b**) PYR13TFSI, (**c**) LiTFSI, and (**d**) D-α-tocopherol polyethylene glycol 1000 succinate (TPGS).

PVDF–HFP Mw = 455,000 g·mol^−1^ (CAS Number 9011-170) from Sigma-Aldrich (Sigma-Aldrich, St. Louis, MO, USA) was used to prepare the composites. LiTFSI, from Aldrich and neat sepiolite, kindly supplied by TOLSA S.A. (TOLSA, Madrid, Spain), were dried under vacuum for 24 h. D-α-tocopherol polyethylene glycol 1000 succinate (TPGS), used to prepare the modified sepiolite TPGS–S, was purchased from Aldrich and used as received. The *N*-Propyl-*N*-methylpyrrolidinium bis(trifluoromethanesulfonyl) imide (PYR13TFSI) employed to prepare the electrolytes was purchased from Solvionic (Solvionic, Toulouse, France). All chemical structures appear in Scheme 1.

### 2.2. Processing

**Scheme 2:**
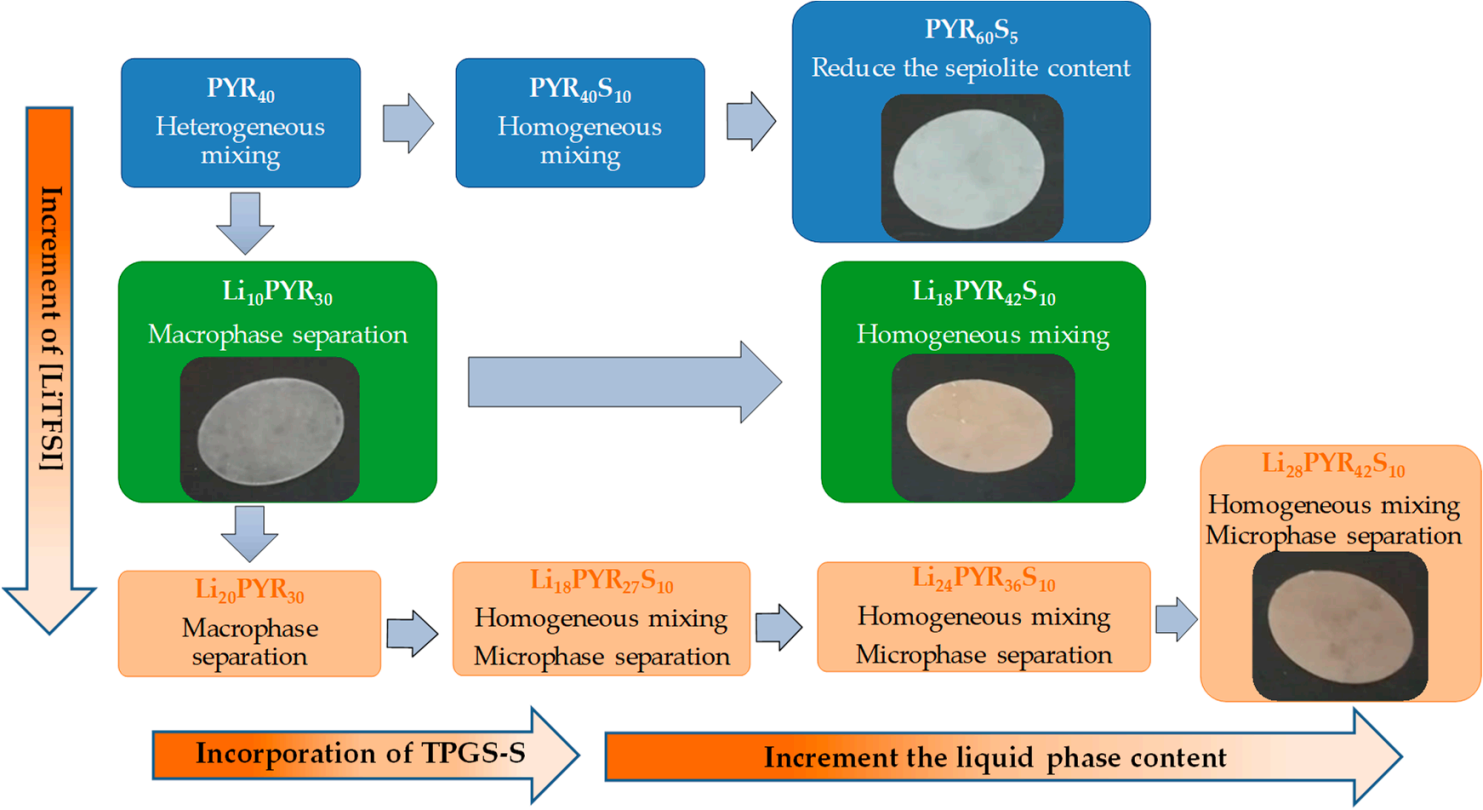
Methodology employed to prepare the set of electrolytes studied in this work. Some pictures showing the appearance of selected samples are included.

The components were melt-compounded in a Haake MiniLab extruder (Thermo Fisher Scientific, Waltham, MA, USA) at 80 rpm, at residence time of 15 min and temperature of 200 °C. To better understand the compatibility in these multicomponent materials, a methodology as depicted in Scheme 2 has been followed. The actual formulation of the electrolytes in Scheme 2 appears in Table 1. The nomenclature of the samples takes into account the weight percentage of each component in the final formulation (PVDF–HFP weight percent is not included for the sake of simplicity), and it appears as a subindex of the component, e.g., Li_18_PYR_42_S_10_ is an electrolyte prepared with 18 wt % of LiTFSI, 42 wt % of PYR13TFSI, 10 wt % of TPGS-S, and 30 wt % of PVDF–HFP.

In Scheme 2, some comments on the appearance of the electrolytes are included. The segregation of a liquid phase in the melt-compounding process that is detected by naked eye is called a macro-phase separation. When the sample looks homogeneous to the naked eye but microscopic domains of different compositions are detected during characterization, it is called a micro-phase separation.

TPGS-S, prepared as detailed elsewhere [25], has been used as stabilizer of the liquid phase and compatibilizer of the final blend. In some of the samples, there is a certain deviation between the nominal and real composition because of liquid phase exudation. The FTIR in Figure S1 shows the IR spectra of all the electrolytes between 700 and 1500 cm^−1^. The 1300–1500 cm^−1^ region reflects well the electrolytes’ composition, as the bands at 1300–1350 cm^−1^ belong to the liquid phase and the ones at 1400 cm^−1^ to the polymer.

### 2.3. Characterization

Characterization of the electrolytes was done on 500-μm film with a controlled thickness, processed by compression molding at 200 °C, for 2 min without pressure and another 3 min at 1.5 bar.

Scanning electron microscopy (SEM) was performed with a PHILIPS XL30 (Philips, Amsterdam, The Netherlands). Samples were fractured after immersion in liquid nitrogen, and the sections were metalized and observed.

The AFM images were obtained on the surface of the electrolytes films with a Veeco Multimode scanning probe microscope equipped with a Nanoscope IV, a controller operating in tapping mode with a phosphorus-doped silicon cantilever (model RTESP).

Differential scanning calorimetry (DSC) studies were performed in a Perkin-Elmer DSC7 (TA Instruments, New Castle, DE, USA). The heat flow was recorded during two cooling–heating cycles at 10 °C·min^−1^ from 220 °C to −80 °C.

ATR-FTIR: IR spectra were recorded on the surface of the electrolytes using a FTIR Perkin-Elmer Spectrum-One (PerkinElmer, Waltham, MA, USA), with 4 scans and 4 cm^−1^ resolution.

Conductivity of the electrolytes was determined in a NOVOCONTROL Concept 40 broadband dielectric spectrometer (Novocontrol Technologies GmbH, Montabaur, Germany) in the temperature range −50 °C to 90 °C and in the frequency range 0.1 and 10^7^ Hz. Disk films of dimensions of 2 cm diameter and ~500 μm thickness were inserted between two gold-plated flat electrodes, then a frequency sweep was done every 10 °C, cooling to −50 °C and then heating to 90 °C; thereafter, the same measurements were done but cooling from 90 to 25 °C. σ of the samples was calculated by using conventional methods based on the Nyquist diagram and the phase angle as a function of the frequency plot, as described before [9].

Determination of diffusion coefficients (D) was done by ^7^Li and ^19^F PFG-NMR in a Bruker AvanceTM 400 spectrometer (Bruker BioSpin GmbH, Rheinstetten, Germany) equipped with a 89 mm wide bore, 9.4 T superconducting magnet (Larmor frequencies of ^7^Li and ^19^F at 155.51 and 376.51 MHz, respectively). The ^7^Li and ^19^F diffusion data were acquired at 25 ± 0.1 °C with a Bruker diffusion probe head, Diff60, using 90° radiofrequency (rf) pulse lengths of 11.0 μs.

The mechanical properties of the dumbbell specimen were determined using a universal tensile machine (MTS model DX 2000, Eden Prairie, MN, USA) with crosshead speed of 10 mm·min^−1^. The sample dimensions were 2 mm × 15 mm. The results were computed from the stress–strain curve calculating the average of three measurement values.

## 3. Results and Discussion

To the best of our knowledge, melt-compounding has never been used to prepare PVDF–HFP electrolytes, though it is by far the most sustainable and scalable procedure for the preparation of materials. This work is devoted to the systematic study of the structure and final properties of a very complete set of electrolytes, which have been done following the methodology illustrated in Scheme 2. The set of electrolytes collected in Table 1 were prepared by increasing progressively the liquid phase fraction, which comprises only PYR13TFSI or a solution of LiTFSI in PYR13TFSI of medium (25–30 wt %) or high (>40 wt %) concentration. When required, TPGS-S is introduced as a compatibilizer and stabilizer of the liquid fraction.

### 3.1. Miscibility/Compatibility of PVDF–HFP with PYR13TFSI and LiTFSI/PYR13TFSI: Phase Separation into Polymer Rich and Polymer Poor Phases as Seen by AFM and DSC

**Figure 1:**
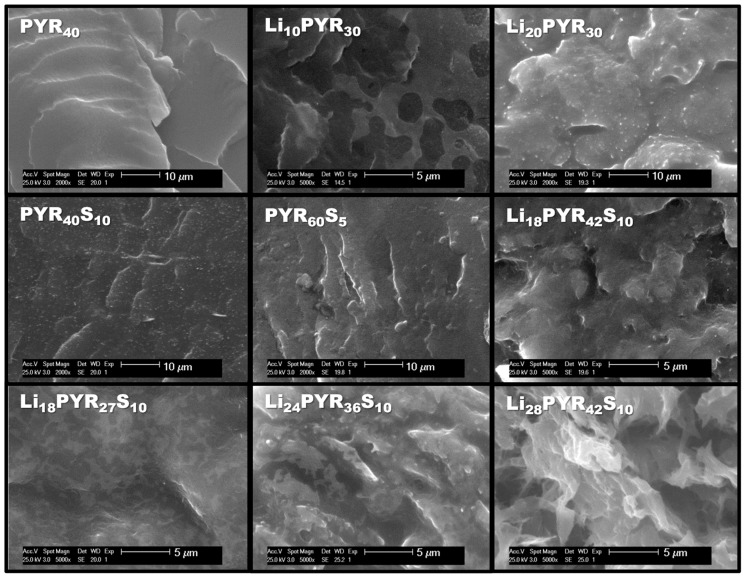
SEM images of all the samples in Table 1.

Blends of PVDF–HFP with PYR13TFSI alone do not look macroscopically phase segregated even at high liquid fractions (PYR_40_). This is illustrated by the photographs in Scheme 2 and the PYR_40_ SEM image in Figure 1. A high degree of mixing is evidenced by the strong decrease in the PVDF–HFP Tc and Tg (Table 1). However, when instead of PYR13TFSI, a dissolution of LiTFSI in PYR13TFSI is employed, macroscopic phase separation is seen, as an exudation of the liquid phase (Li_10_PYR_30_, Li_20_PYR_30_), so that it is not possible to prepare electrolytes with enough liquid phase to meet minimum electrochemical requirements. The FTIR in Figure S1 in the 1300–1500 cm^−1^ reflect the actual composition of the electrolytes, and it can be seen that electrolytes Li_10_PYR_30_ and Li_20_PYR_30_, without TPGS-S, have a real liquid phase fraction slightly lower to the nominal one.

In previous PEO based electrolytes [8,9] we had already seen strong phase separation between the ethylene carbonate/LiTf liquid phase and the PEO. This macrophase separation turned into a microphase separation (highly enhancing conductivity and very stable along time) when TPGS-S was employed, because of its behavior as a pickering emulsifier of the liquid phase [25]. On the opposite, the good compatibility between many ionic liquids and PEO renders more homogeneous blends. The homogeneous mixing of PEO with imidazolium and pirrolidinium ionic liquids, in combination with the higher viscosity of these ionic liquids as compared to ethylene carbonate, makes PEO/RTIL electrolytes significantly less conductive that PEO/EC ones [10]. In the search of thermoplastic electrolytes that are able to withstand high voltages, safe and secure, and reasonably conducting, we are testing that same phase-separated morphology strategy with PVDF–HFP as polymer matrix. A set of PVDF–HFP electrolytes, with and without LiTFSI, was prepared incorporating a small fraction of TPGS-S to study its effect on phase separation. It is visually evident that phase separation is much reduced when using TPGS-S, in electrolytes without LiTFSI (PYR_40_S_10_, PYR_60_S_5_) but especially in electrolytes with LiTFSI (Li_18_PYR_27_S_10_, Li_18_PYR_42_S_10_, Li_24_PYR_36_S_10_, Li_28_PYR_42_S_10_). Even in those with large liquid fractions, weight loss by exudation along time is under 0.3% for time periods of about one month (see Figure S2).

Figure 1 shows the morphology of the electrolytes as seen by SEM. The SEM image of PYR_40_S_10_ shows the excellent dispersion of TPGS-S in these electrolytes. TPGS-S is not seen in electrolytes with LiTFSI and/or higher liquid fraction because of the contrast in the images and the excellent compatibility of the fiber within the material. SEM images also suggest that electrolytes with liquid fractions as high as Li_24_PYR_36_S_10_ are non porous, i.e., that they are dense electrolytes. In many of these electrolytes (Li_10_PYR_30_, Li_18_PYR_27_S_10_, Li_24_PYR_36_S_10_), an interesting pattern of darker and lighter regions is well seen. This pattern is composed of large, dark domains in Li_10_PYR_30_, which are much smaller in Li_18_PYR_27_S_10_ and Li_24_PYR_36_S_10_. Li_28_PYR_42_S_10_, with a composition which is very similar to that employed in solvent cast PVDF–HFP electrolytes with LiTFSI and BMPTFSI [24], shows the dark and light pattern too.

**Figure 2:**
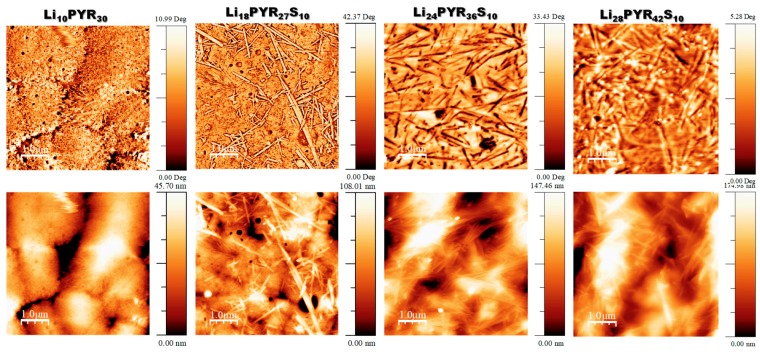
Phase and height AFM images of some of the electrolytes.

On their turn, the AFM images in Figure 2 show the coexistence of hard and soft regions in electrolyte Li_10_PYR_30_, probably polymer-rich crystalline regions (hard) and polymer-poor amorphous regions (soft). In Li_10_PYR_30_, crystalline regions adopt a spherulitic morphology, while in Li_18_PYR_27_S_10_ it becomes more difficult to individualize large crystalline regions. Contrary to what happens in SEM images, TPGS-S is also well seen in those electrolytes which contain it (Li_18_PYR_27_S_10_, Li_24_PYR_36_S_10_ and Li_28_PYR_42_S_10_), as long, hard, well dispersed and abundant fibers. The electrolytes with the largest liquid phase fraction and the lowest crystalline fraction (Li_24_PYR_36_S_10_ and Li_28_PYR_42_S_10_) show no crystalline hard regions, but rather a uniform amorphous matrix in which again the TPGS-S fibers are well seen.

**Figure 3:**
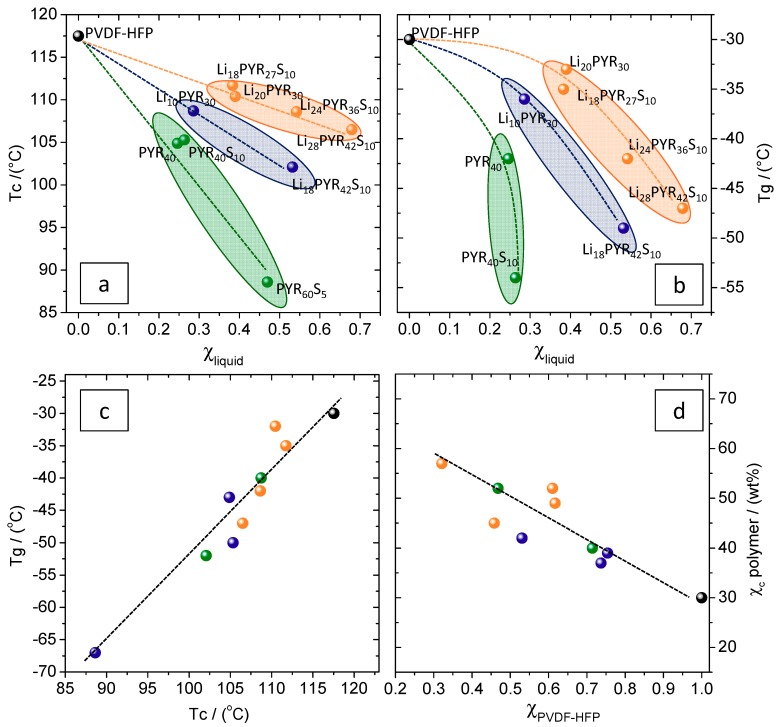
(**a**) Tc as a function of the molar fraction of liquid phase (χ_liquid_); (**b**) Tg as a function of the molar fraction of liquid phase; (**c**) concomitant variation of Tg and Tc; and (**d**) crystalline fraction in the polymer (X_c_) as a function of the molar fraction of PVDF–HFP in the electrolyte (χ_PVDF_–_HFP_). Electrolytes without LiTFSI appear in green, electrolytes with 25–30 wt % of LiTFSI in PYR13TFSI in blue and electrolytes with over 40 wt % of LiTFSI in PYR13TFSI appear in orange. The dash lines in (**a**,**b**) are guides for the eyes, and in (**c**,**d**) are linear fits.

Table 1 shows the Tc and Tg of the different electrolytes. The blending of PVDF–HFP with PYR13TFSI in the absence of TPGS-S (PYR_40_) decreases strongly the polymer’s Tc and Tg, suggesting that these two components are miscible to a significant degree. Incorporation of LiTFSI (in the absence of TPGS-S) (Li_10_PYR_30_, Li_20_PYR_30_) also produces a certain decrease of Tc and of Tg, but to a lower extent than in PYR_40_. Figure S3a shows the very remarkable decrease on the Tc in all the electrolytes. Figure 3a,b shows the variation of the polymer’s Tc and Tg as a function of the liquid fraction (PYR13TFSI or LiTFSI + PYR13TFSI) in all the electrolytes. Three different trends seem to exist in the Tc and Tg depression, depending on the fraction of LiTFSI in the liquid phase. Those electrolytes prepared without LiTFSI (PYR_40_, PYR_40_S_10_, PYR_60_S_5_, in green in Figure 3) depress much more both Tc and Tg; those prepared with a 50 wt % of LiTFSI in PYR13TFSI (Li_20_PYR_30_, Li_18_PYR_27_S_10_, Li_24_PYR_36_S_10_, and Li_28_PYR_42_S_10_, in orange in Figure 3) depress both Tc and Tg much less, and those prepared with a 35 wt % of LiTFSI in PYR13TFSI (Li_10_PYR_30_, Li_18_PYR_42_S_10_, in blue in Figure 3) are intermediate. When studying the DSC of these electrolytes, it has to be taken into account that their phase distribution is very complex, and this reflects itself in DSC scans with multiple steps and endotherms, as shown in Figure S3b where the first DSC scan of all the electrolytes appear. These DSC include the Tg of the polymer blended with different fractions of PYR13TFSI or LiTFSI/PYR13TFSI in the low temperature range and the melting of the liquid phase close to 10 °C.

That the decrease in Tg and Tc occurs not only as a function of the liquid phase fraction, but also as a function of the LiTFSI content is not surprising. On one hand, the progressive increase of PYR13TFSI is plasticizing the polymer and lowering its viscosity, but on the other, the solutions of LiTFSI in PYR13TFSI are much less miscible with PVDF–HFP than pure PYR13TFSI alone (even with the help of TPGS-S). Microphase separation into polymer-rich and polymer-poor domains is more conspicuous, then, as the LiTFSI content increases, the effect of the liquid phase on the polymer’s transitions and relaxations lowers. See how in Figure 3a,b three different trends are seen, depending on whether the electrolyte has no LiTFSI (green), or it has about 25–30 wt % (blue) or over 40 wt % (orange) of LiTFSI in the liquid phase. Polymer-poor phases consist of blends of PYR13TFSI /LiTFSI with the amorphous fraction of PVDF–HFP while polymer-rich phases are mostly crystalline. The connection between Tc and Tg is well seen in Figure 3c.

### 3.2. Crystalline Phase of PVDF–HFP in the Electrolytes

The PVDF–HFP electrolytes presented in this work contain always a certain amount of crystalline fraction of PVDF–HFP (Table 1). Figure 3d shows that as the molar fraction of the polymer decreases in the electrolyte, a strong increase of the PVDF–HFP crystallinity is very clearly seen. Figure S1a shows the 700 cm^−1^ to 900 cm^−1^ region, which contains IR vibrations sensitive to the conformation of the polymer backbone. It can be seen how the bands at 761 and 795 cm^−1^ corresponding to conformations in the α PVDF–HFP crystalline phase, the most stable thermodynamically, disappear, while the band at 840 cm^−1^ which corresponds to the β PVDF–HFP phase increases. Then, blending with the ionic liquid makes the crystalline fraction become higher and dominated by the β phase. This effect of the presence of LiTFSI on the crystallization of PVDF–HFP has been reported before and attributed to the increasing polarity of the medium [26].

**Figure 4:**
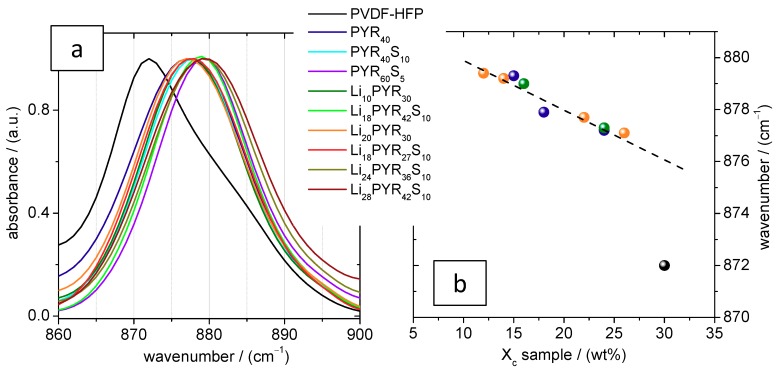
(**a**) The normalized PVDF–HFP 872 cm^−1^ band in the copolymer and its progressive shifting in all the electrolytes and (**b**) shift of the 872 cm^−1^ band towards 880 cm^−1^ with increasing crystalline fraction in the whole sample (X_c_ sample).

Figure 4a shows the 870–890 cm^−1^ IR region. In PVDF–HFP, this region is composed of a band at 872 cm^−1^, and a shoulder at 880 cm^−1^ which can belong to any of the crystalline phases or to the amorphous phase, for it appears in dissolved PVDF–HFP (Figure S4) [26]. The peak, which is at 872 cm^−1^ in the copolymer, seems to shift towards 880 cm^−1^ as the liquid fraction in the electrolyte increases and the crystalline phase increases and changes from α to β. In fact, there is a linear relationship between the position of the band and the overall molar fraction of crystalline PVDF–HFP in the electrolyte (X_c_ sample). There is no such relationship between the position of the band and the wt % of crystalline fraction in the polymer (X_c_ polymer), so it does not seem to be related directly with the fraction of β phase in the PVDF–HFP, but rather to the increasing polarity of the environment, because of the increasing fraction of β crystals and of ionic liquid.

### 3.3. Mechanical Properties

Since the recent discovery on the effect of the electrolytes’ elastic modulus on the metallic dendrite growth detailed in the Section 1, Introduction, mechanical properties’ importance has grown beyond their influence of the electrolyte’s dimensional stability, and so we have undertaken a complete stress–strain mechanical study of the samples in this work. Actual stress–strain curves appear in Figure S5, while elastic modulus and elongation at break are listed in Table 1. When analyzing those results, it is important to note that the small fraction of crystalline PVDF–HFP in the electrolyte is, together with the TPGS-S, the only solid fraction of the formulation at room temperature, as the glass transition of PVDF–HFP occurs at very low temperatures. This solid fraction and its distribution (phase morphology) are going to be responsible not only for the dimensional stability and toughness of the electrolyte, but also for impeding Li dendritic growth.

The stress–strain curves of all electrolytes (Figure S5) are typical of a thermoplastic polymer. There is a strong decrease in elastic modulus when comparing PVDF–HFP with the electrolyte with the lowest fraction of liquid phase, PYR_40_. Then, as the X_c_ in the sample decreases from PYR_40_ (or conversely as the liquid fraction increases), the elastic modulus is progressively reduced (Table 1), The decrease of the elastic modulus is gentle, what allows for the electrolytes with very low polymer fraction (at or under 30 wt %) to keep reasonable elastic modulus, making them easy to handle. Concomitant to the initial abrupt decrease in elastic modulus, the elongation at break (Table 1) increases substantially when the polymer is plasticised, being the most deformable Li_10_PYR_30_. However, the continuous dilution of the polymer phase makes the elongation at break to begin decreasing at a given point, very possibly as a consequence of the entanglement dilution.

### 3.4. Conductivity and Ion Diffusivity

**Figure 5:**
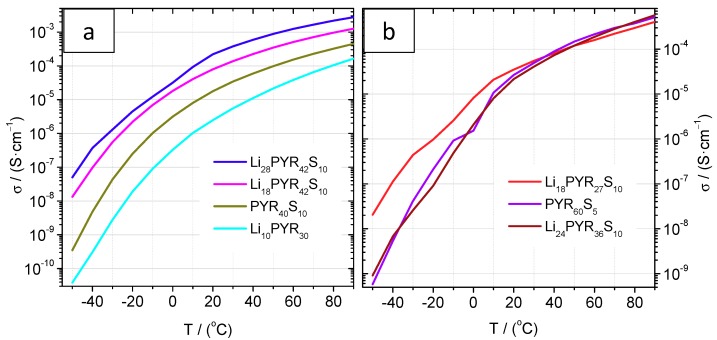
(**a**) σ(T) of electrolytes PYR_40_S_10_, Li_10_PYR_30_, Li_18_PYR_42_S_10_, and Li_28_PYR_42_S_10_ and (**b**) σ(T) of electrolytes Li_18_PYR_27_S_10_, PYR_60_S_5_, and Li_24_PYR_36_S_10_.

Figure 5 collects the σ of the electrolytes as a function of temperature, on heating from −50 to 90 °C. For the sake of clarity, the electrolytes have been divided into two groups, the first one comprises electrolytes which show little or no step-like variations in σ(T) (Figure 5a), while the second contains the electrolytes where significant step-like variations in σ(T) are seen (Figure 5b). These step-like variations often appear in σ(T) curves and are directly related to phase transitions or relaxations which involve important mobility changes, such as glass transition or melting.

Good correlation is seen between the steps in σ(T) in Figure 5b and the transitions and relaxations under room temperature shown in the DSC in Figure S3b. In Li_24_PYR_36_S_10_, a conspicuous Tg appears in the low T region (≈−40 °C) followed by a series of steps between −30 °C and −10 °C and also close to 10 °C. The reflection of these relaxations and melting endotherms (≈10 °C) on mobility is well seen in the step-like σ(T) of this electrolyte. In Li_18_PYR_27_S_10_ and PYR_60_S_5_, melting endotherms and relaxations appear in the temperature range −20 °C to 20 °C, which also have a clear effect on σ(T) (Figure 5b). Also, Li_28_PYR_42_S_10_ shows a Tg under −40 °C and several small specific heat steps up to 20 °C, indicating that domains with different composition and mobility exist in this sample, the motions of which onset at different temperatures and show themselves as a small step in σ(T) (Figure 5a) at about 10 °C (and maybe also at −50 °C). As a general rule, more phase separation is seen when increasing the [LiTFSI] or decreasing the TPGS-S wt %, because the incorporation of LiTFSI makes the ionic liquid less compatible with PVDF–HFP, while TGPS-S stabilizes the liquid phase.

It is noteworthy that the conspicuous α relaxation at 50 °C (Figure S3b) of electrolytes Li_18_PYR_27_S_10_, PYR_60_S_5_, Li_10_PYR_30_, PYR_40_S_10_, and Li_18_PYR_42_S_10_, has no effect on σ(T). Thus, this relaxation, which has been related to the crystalline interfacial dynamics of PVDF–HFP [27], seems to have no effect on the ionic or molecular mobility of these electrolytes. Then, over room temperature none of the electrolytes suffers step-like variation of σ, and hence a comparison of σ of the different electrolytes at 25 °C makes sense. This appears in Table 2, together with conductivity at 75 °C for all the electrolytes. σ at 25 °C ranges from 7 × 10^–4^ mS·cm^−1^ the lowest to 0.2 mS·cm^−1^ the highest, which is a difference of three orders of magnitude roughly.

**Figure 6:**
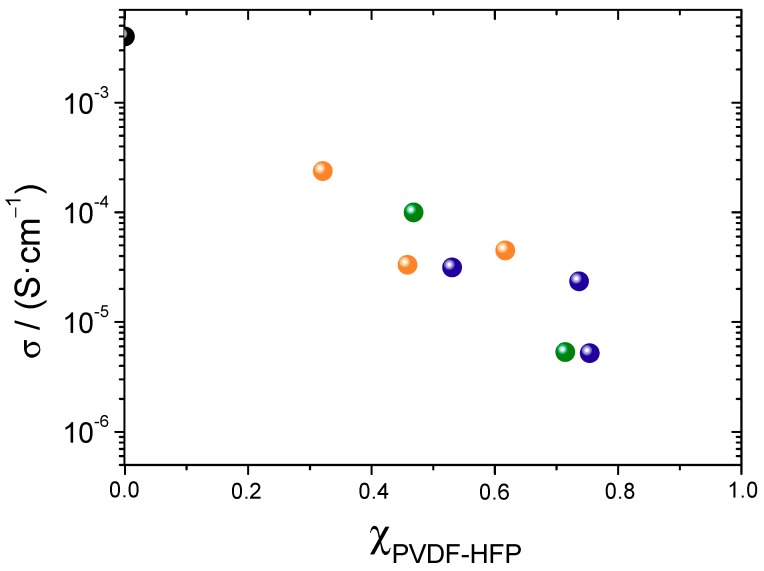
σ as a fraction of the molar fraction of PVDF–HFP (χ_PVDF_–_HFP_) in the electrolyte at 25 °C.

Figure 6 represents σ (25 °C) as a function of the molar fraction of PVDF–HFP (χ_PVDF_–_HFP_) in the electrolyte. The value of σ (25 °C) of pure PYR13TFSI has been included (in black). As the composition of the electrolytes becomes richer in liquid phase, their σ increases logarithmically. In fact, the σ of Li_28_PYR_42_S_10_ is very remarkable and comparable to that obtained in electrolytes prepared with PVDF–HFP and PYR14TFSI/LiTFSI (not PYR13TFSI) by solvent casting [24]. The logarithmic decrease of σ with χ_PVDF_–_HFP_ shown in Figure 6 suggests that further decreases in the polymer fraction may produce substantial increases in σ.

**Figure 7:**
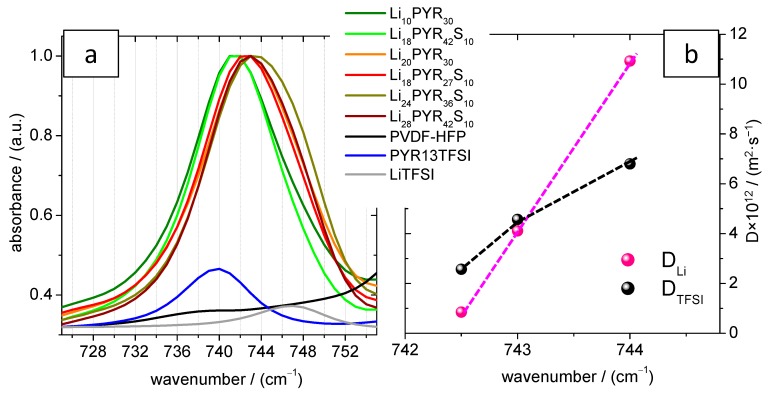
(**a**) The normalized υ(S-N) band of TFSI in all the electrolytes together with the same band in PYR13TFSI and LiTFSI (this spectral region for neat PVDF–HFP is also included), and (**b**) *D_Li_* and *D_TFSI_* at 25 °C as a function of the position of the υ(S-N) in Li_18_PYR_27_S_10_, Li_24_PYR_36_S_10_, and LI_28_PYR_42_S_10_.

Searching for the likely occurrence of anion exchange mechanism of Li diffusion [28,29,30], the electrolytes with higher content of LiTFSI were prepared (Li_24_PYR_36_S_10_ and Li_28_PYR_42_S_10_), and the ion diffusion of Li and TFSI was studied by PFG-NMR. The diffusion coefficients of Li (*D_Li_*) and TFSI (*D_TFSI_*) appear in Table 2. As reported previously [28,29,30], over a certain [LiTFSI] threshold, *D_Li_* increases well over *D_TFSI_*. While in Li_18_PYR_27_S_10_, $\frac{D_{Li}}{D_{TFSI}}=$ 0.31, which is a value typical of viscosity governed transport, in Li_28_PYR_42_S_10_, $\frac{D_{Li}}{D_{TFSI}}=0.9$ and in Li_24_PYR_36_S_10_, $\frac{D_{Li}}{D_{TFSI}}=1.6$. The $\frac{D_{Li}}{D_{TFSI}}$ increase on going from Li_18_PYR_27_S_10_ to the other two is easy to understand, because of the [LiTFSI] increase, but both Li_24_PYR_36_S_10_ and Li_28_PYR_42_S_10_ have similar LiTFSI content. Figure 7a shows the IR spectra of the υ(S-N) region of TFSI, which is related to the aggregation state of the anions [31]. The υ(S-N) of the TFSI band in PYR13TFSI appears at 740 cm^−1^, while that of TFSI in LiTFSI appears at 747 cm^−1^. The first one corresponds to a “free” TFSI while the other corresponds to a more “bound” TFSI. In the set of IR which appears in Figure 7a, Li_28_PYR_42_S_10_ would have the less bound TFSI, while Li_24_PYR_36_S_10_ would be clearly the more strongly bound TFSI, for its υ(S-N) band is the closest to 747 cm^−1^. Thus, the TFSI in Li_24_PYR_36_S_10_ is more strongly bound to the Li cation than in any other of the electrolytes. Figure 7b correlates the shift in the υ(S-N) IR band with the increase of *D_Li_* relative to *D_TFSI_*.

The well-known Nernst–Einstein equation:

(1) $$\sigma_{NE}=\frac{F^{2}}{RT}\cdot \underset{i}{\sum }n_{i}\alpha_{i}\cdot D_{i}$$

has been used to calculate *σ_NE_*, global and for Li ion, by making use of the diffusivity and molar concentration of the ions in Table 1 and Table 2, respectively, and assuming the ionicity α to be 1. These values appear in columns 6 and 7 of Table 2. To do so, D_PYR_, which cannot be measured in our NMR facilities, has been estimated to be the same as *D_TFSI_*. Once the *σ_NE_* is obtained, it is possible to calculate the ionicity $\alpha =\frac{\sigma }{\sigma_{NE}}$, which values appear in column 8 of Table 2. Interestingly, while Li_24_PYR_36_S_10_ has the largest *D_Li_* by far, and apparently could be the most interesting electrolyte for a Li battery, its experimental *σ* is very low, and consequently ionicity *α* is also very low. For some reason, there is a large difference between the local picture provided by diffusivity, and the macroscopic behavior of conductivity of this electrolyte. On the opposite, Li_28_PYR_42_S_10_ shows an *α* which is reasonably high, implying that the actual *σ* of Li may be higher in Li_28_PYR_42_S_10_ than in Li_24_PYR_36_S_10_. In any case, both electrolytes are very promising because of their overall *σ*, *D_Li_* and mechanical properties and deserve further electrochemical studies.

## 4. Conclusions

With the electrochemically stable PVDF–HFP, pyrrolidinium based ionic liquids, and LiTFSI, it is possible to prepare by melt-compounding electrolytes, which have ionic conductivity very similar to those prepared by solvent casting, with the advantage that the solvent-free extrusion procedure takes several minutes and scales industrially easily, while solvent casting not only is less sustainable but also much more time consuming and difficult to scale up. The preparation of these extruded electrolytes requires employing TPGS-S as blend compatibilizer, especially when solutions of LiTFSI in PYR13TFSI are employed. The mechanical properties of these electrolytes prove that they behave as typical thermoplastic polymers, their elastic modulus being dependent on the liquid fraction incorporated. The crystallization temperature and the glass transition of PVDF–HFP become highly depressed in these materials, and they are a function of the liquid fraction in the electrolyte. Both mechanical and thermal properties suggest that most of them are dense membranes with a microphase separation, rather than porous membranes soaked in a liquid, which makes them appealing as Li dendrite barriers. Some of these electrolytes have been prepared with [LiTFSI] sufficiently high as to see the anion exchange Li transport mechanism, what makes Li ion diffusivity very high and comparable or even greater than TFSI diffusivity.

## Figures and Tables

**Table 1:** Formulation; crystallinity (in wt %) referred to the sample, X_c_ sample, and to the PVDF–HFP, X_c_ polymer; thermal properties (crystallization temperature, Tc; glass transition temperature, Tg) and mechanical properties (Young´s modulus, E, and elongation at break).

Sample	PVDF–HFP (mol m^−3^)	PYR13TFSI (mol m^−3^)	TPGS-S (wt %)	LiTFSI (mol m^−3^)	X_c_ (wt %) Sample	X_c_ (wt %) Polymer	Tc (°C)	Tg (°C)	E (Mpa)	Elongation at Break (%)
PVDF–HFP					30	30	117.5	−30	398.5	344.7
PYR_40_	4600	1500	0	0,0	24	39	104.9	−43	19.6	556.4
Li_10_PYR_30_	4500	1200	0	600	24	40	108.7	−39	128.2	677.2
Li _20_PYR_30_	3600	1200	0	1100	26	52	110.4	−32	187.1	595.1
PYR_40_S_10_	4200	1500	10	0	18	37	105.3	−50	71.1	530.2
PYR_60_S_5_	2600	2300	5	0	15	42	88.6	*-*	57	21.5
Li_18_PYR_27_S_10_	2900	900	10	900	22	49	111.7	−35	107.5	404.6
Li_18_PYR_42_S_10_	2200	1600	10	900	16	52	102.1	−52	39.7	46.4
Li_24_PYR_36_S_10_	2200	1300	10	1300	14	45	108.6	−42	67.4	13.0
Li_28_PYR_42_S_10_	1390	1470	10	1470	12	57	106.5	−47	49.3	34.9

**Table 2:** Conductivity at 25 °C, σ (25 °C), and 75 °C, σ (75 °C), and ion diffusivity of the electrolytes. Nernst–Einstein conductivity (*σ_NE_*) calculated from Equation (1) and ionicity (α).

Sample	σ (mS·cm^−1^)		Diffusion (25 °C) 10^12^ (m^2^·s^−1^)		*σ_NE_* (mS·cm^−1^)		α
	σ (25 °C)	σ (75 °C)	*D_Li_*	*D_TFSI_*	Global	Li	
PYR_40_	5 × 10^−3^	-	-	-	-	-	-
Li_10_PYR_30_	5 × 10^−3^	0.09	-	-	-	-	-
Li_20_PYR_30_	7 × 10^−4^	-	-	-	-	-	-
PYR_40_S_10_	0.02	0.28	-	-	-	-	-
PYR_60_S_10_	0.03	0.34	-	-	-	-	-
Li_18_PYR_27_S_10_	0.05	0.26	0.8	2.6	0.29	0.03	0.16
Li_18_PYR_42_S_10_	0.10	0.82	-	-	-	-	-
Li_24_PYR_36_S_10_	0.03	0.35	10.9	6.8	1.52	0.52	0.02
Li_28_PYR_42_S_10_	0.24	1.81	4.1	4.6	0.98	0.22	0.24

## Supplementary Materials

The following are available online at https://www.mdpi.com/2077-0375/9/4/50/s1, Figure S1: FTIR spectra in the 700 to 1500 cm^−1^ region illustrating the crystallization forms of polyvinylidene fluoride–hexafluoropropylene (PVDF–HFP) and the electrolyte composition. Figure S2: Gravimetric determination of the liquid phase loss in two electrolytes along time. Figure S3: (a) Differential Scanning Calorimetry (DSC) on cooling at 10 °C·min^−1^ showing the crystallization of the electrolytes and (b) DSC on heating at 10 °C·min^−1^ showing the Tg and Tm of the electrolytes, divided into two groups for better visualization. Figure S4: FTIR spectrum of PVDF–HFP in DMF solution, 1 wt %. Figure S5: Strain–stress curves of all the electrolytes. They have been divided into two groups for better visualization.
