# Supplementary material for: Solvent-Free and Scalable Procedure to Prepare PYR13TFSI/LiTFSI/PVDF–HFP Thermoplastic Electrolytes with Controlled Phase Separation and Enhanced Li Ion Diffusion
Source: Membranes (Basel). 2019 Apr 10;9(4):50. doi: 10.3390/membranes9040050 (PMC6523596; doi:10.3390/membranes9040050)
Supplement: Supplementary file 1 [file membranes-09-00050-s001.pdf]

Supplementary Material

Solvent-free and Scalable Procedure to Prepare PYR13TFSI/LiTFSI/PVDF–HFP Thermoplastic Electrolytes with Controlled Phase Separation and Enhanced Li Ion Diffusion

Víctor Gregorio, Nuria García and Pilar Tiemblo *

Instituto de Ciencia y Tecnología de Polímeros, ICTP-CSIC, Juan de la Cierva 3, 28006 Madrid, Spain; v.gregorio@ictp.csic.es (V.G.); ngarcia@ictp.csic.es (N.G.)

* Correspondence: ptiemblo@ictp.csic.es

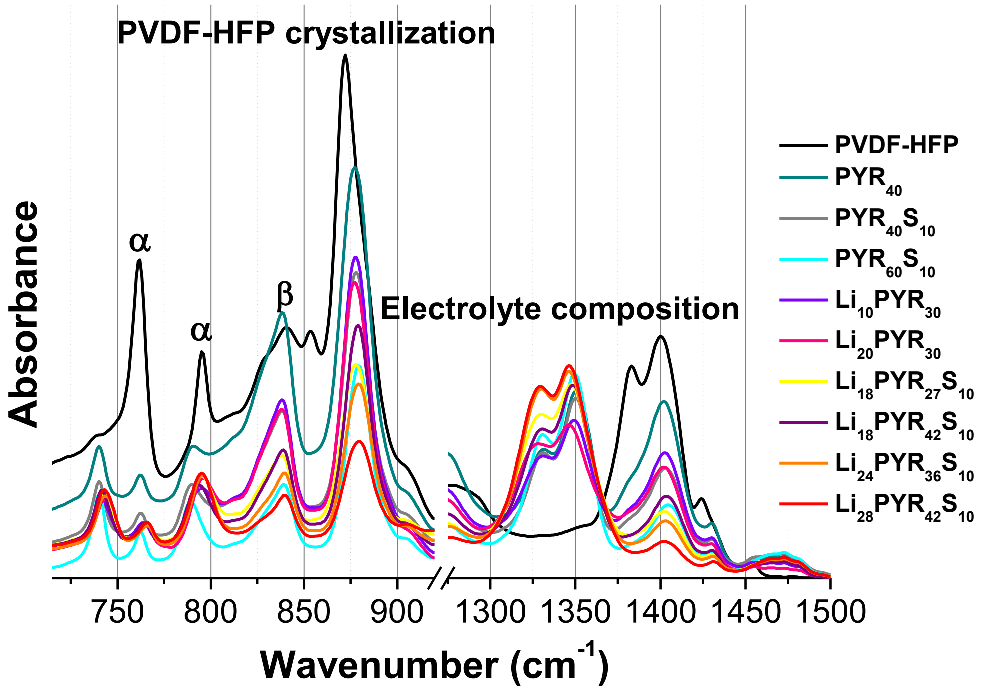

Figure S1. FTIR spectra in the 700 to 1500 cm^{-1} region illustrating the crystallization forms of polyvinylidene fluoride–hexafluoropropylene (PVDF–HFP) and the electrolyte composition.

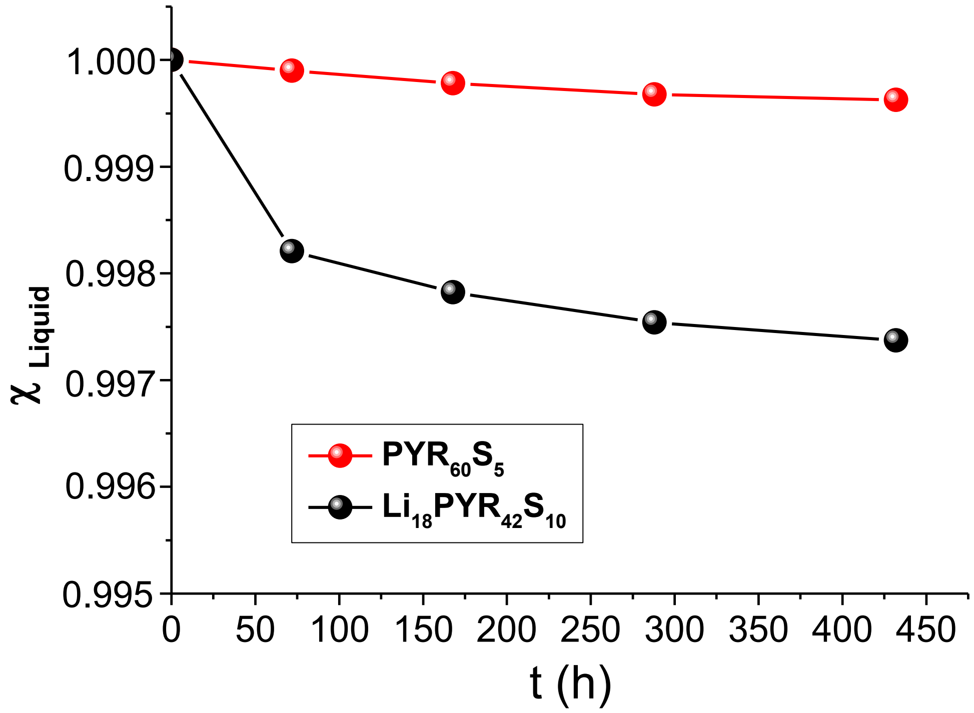

Figure S2. Gravimetric determination of the liquid phase loss in two electrolytes along time.

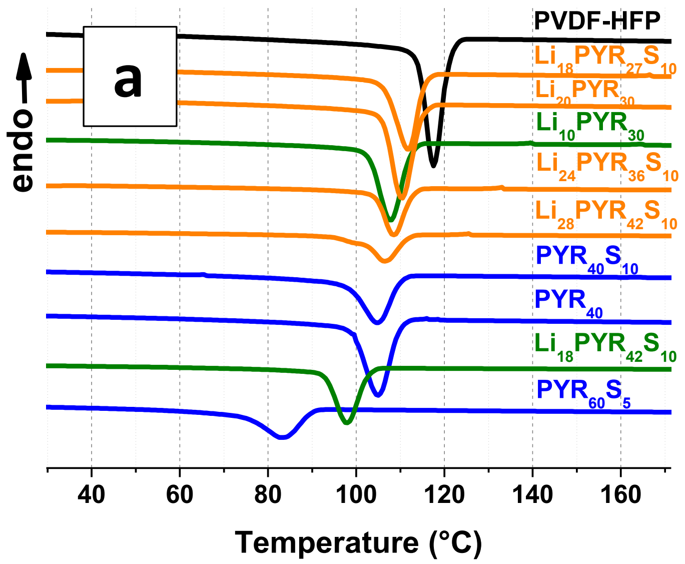

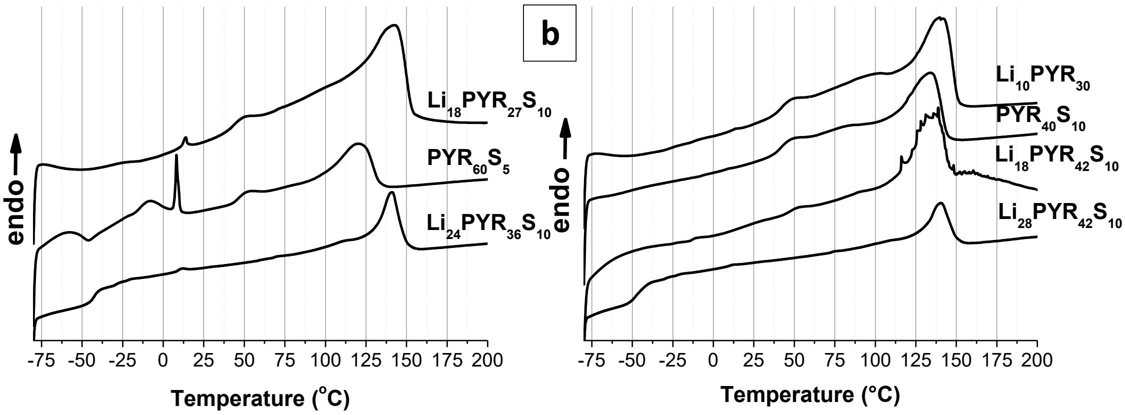

Figure S3. (**a**) Differential scanning calorimetry (DSC) on cooling at 10 °C·min^{-1} showing the crystallization of the electrolytes (**b**) DSC on heating at 10 °C·min^{-1} showing the Tg and Tm of the electrolytes, divided into two groups for better visualization.

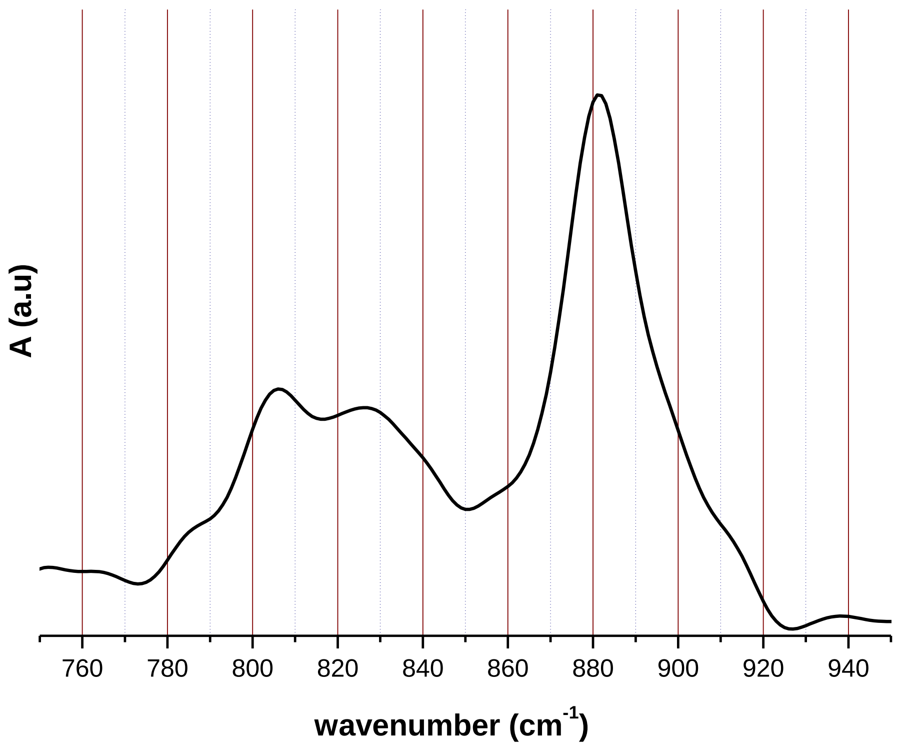

Figure S4: FTIR spectrum of PVDF-HFP in DMF solution, 1 wt %.

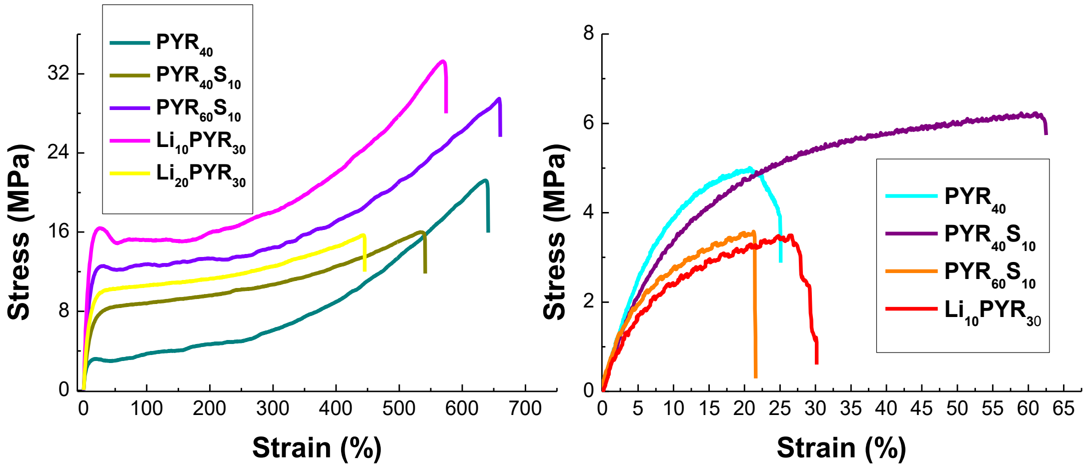

Figure S5. Strain-stress curves of all the electrolytes. They have been divided into two groups for better visualization.
